# Supplementary material for: Are carbon emissions trading and green financial instruments synergistic? -Comprehensive quantitative research based on content analysis
Source: PLoS One. 2024 Mar 7;19(3):e0298601. doi: 10.1371/journal.pone.0298601 (PMC10919601; doi:10.1371/journal.pone.0298601)
Supplement: S1 Data — (ZIP) [file pone.0298601.s001.zip › Data/地级市Z_F工作报告.zip!/╔╜╬≈/╜·│╟╩╨/└┤╫╘╣1⁄2╓┌║┼í╛┬φ┐╦╩2╛▌═°í┐_2018.docx]

各位代表：

　　现在，我代表市人民政府向大会报告工作，请予审议，并请市政协委员和列席人员提出意见。

一、2017年工作回顾

　　一年来，我们以习近平新时代中国特色社会主义思想为指引，深入学习宣传贯彻党的十九大精神，认真落实省委、省政府和市委决策部署，坚定不移践行新发展理念，深入实施转型综改、创新驱动战略，全力做好稳增长、促改革、调结构、惠民生、防风险各项工作，较好地完成了市七届人大二次会议确定的目标任务。全年地区生产总值完成1151.5亿元，增长6.1%；规模以上工业增加值完成468.6亿元，增长5.5%；全社会固定资产投资（新口径）完成430.7亿元，增长6.3%；社会消费品零售总额完成415亿元，增长7.5%；外贸进出口总额完成46.8亿元，增长17.6%；一般公共预算收入首次突破百亿大关，完成101.4亿元，增长13.5%；城镇居民人均可支配收入完成30142元，增长6.8%；农村居民人均可支配收入完成12511元，增长7.5%。

　　（一）产业转型加快推进。编制完成《晋城市发展高端装备制造业总体思路》。兰花己内酰胺项目建成投产，晋煤华昱高硫煤洁净利用项目进入单体试车阶段，中船重工海装风电总装维护基地首台风机具备下线条件，南村绿色智能铸造创新产业园、清慧5000万件轨道交通材料等项目进展顺利。全市战略性新兴产业、非煤产业增加值占规上工业增加值比重分别达到11.3%和37.6%。服务业新业态、新模式不断涌现，占GDP比重达到41.9%。成立了市县两级旅游发展委员会，16家重点景区完成所有权和经营权分离改革，太行一号国家风景道陵川、阳城试验段开工建设，全市实现旅游总收入444.6亿元，增长24.7%，接待游客4848万人次，增长24.8%。成功举办首届现代农业交易博览会，农产品加工企业销售收入完成78.2亿元，增长7%。首家与省科技厅建立厅市工作会商制度，省级以上众创平台达到10家，省级创业基地达到17家。省、市重点工程累计完成投资288.5亿元，完成率103.2%。

　　（二）改革开放持续深化。认真贯彻落实国务院42号文件精神及省委、省政府决策部署，全面系统深化转型综改工作。扎实推进“三去一降一补”，全年共关闭5座煤矿，退出产能725万吨，化解钢铁过剩产能85万吨。率先在全省组建市级国有资本投资运营公司，完成47户市属国有企业的吸收合并和划转。晋城经济技术开发区“三化三制”改革基本完成，扩区方案已获省政府批复。高平经济技术开发区获省政府批准设立。编制完成中国（山西）自由贸易试验区（晋城片区）研究报告。积极申报国家级资源型地区转型发展金融改革创新试验区。扎实开展“1+9”专项行动，全力打造“六最”营商环境。全市域推进县乡医疗卫生机构一体化改革，六县（市、区）全部成立了医疗集团，国务院医改办和省委、省政府给予高度评价。泽州县国家级农村集体经营性建设用地入市试点工作受到国土资源部充分肯定。我市被确定为全省唯一的农林文旅康产业融合发展整市推进试点。全省采煤沉陷区治理现场推进会在我市召开。不动产统一登记、城市管理综合行政执法、投融资、财税体制等改革均取得重大进展。出台招商引资、招才引智“双十条”政策。晋城与郑州、晋城与焦作互通城际公交。成功举办第二届海峡两岸神农炎帝文化旅游招商系列活动、第四届山西（晋城）太行山文化旅游节和首届全域旅游嘉年华活动。

　　（三）城乡环境更加宜居。我市成为山西唯一被列入中原城市群核心发展区的城市，入选全国第二批城市设计试点城市，《美丽晋城战略规划》编制完成。高沁高速、207国道晋城过境段、文博路、书院街、太岳街建成通车，阳蟒高速进展顺利，陵沁路西北环改线、中原街改造及综合管廊建设等重大基础设施项目具备开工条件，太焦高铁加速施工，民航机场选址取得实质性进展。老城更新与保护前期工作扎实推进。以遏制增量和新建道路两侧全面“清零”为突破口，市区“双违”治理攻坚战已经打响。神农镇、巴公镇入选第二批全国特色小镇。认真落实中央、省环保督察反馈意见，一批突出的环境问题得到整改。深化大气污染防治，全市所有火电企业均实现超低排放，集中整治“散乱污”企业866家，淘汰取缔、对标改造和清洁替代燃煤锅炉2247台，实施居民清洁取暖改造11.26万户。四级河长制管理体系初步建立，《沁（丹）河流域生态修复与保护规划》通过省政府批复，沁河综合治理开工建设，丹河湿地入选国家级湿地公园试点，市区10条黑臭水体治理基本完成。扎实推进改善农村人居环境“四大工程”，创建了6个省级、20个市级美丽宜居示范村。完成国省营造林1.17万亩，义务植树300万株，环城生态圈绿化1.3万亩，太行洪谷晋升为国家级森林公园。进入2018-2020年创建周期全国文明城市提名前20名，取得历史最好成绩。

　　（四）民生事业全面进步。全市公共财政民生支出达146.5亿元，占一般公共预算支出的82%。九件民生实事全部落实。陵川、沁水两个贫困县脱贫摘帽通过初审，73个贫困村退出、3.15万贫困人口脱贫，贫困发生率由5.3％降至0.7％。全市城乡义务教育一体化改革启动实施，新（改）建幼儿园15所，凤城中学高中部、市中小学综合实践基地主体工程基本完工，陵川县通过国家义务教育发展基本均衡县认定，市旅游职业教育集团挂牌成立。新开工棚改安置房1853套，基本建成2045套，公租房累计分配8388套。城镇新增就业人数超额完成年度目标，城镇登记失业率控制在1.7%以内。城乡低保、特困人员供养、人均基本公共卫生服务经费等标准都有新提高。成功举办了晋城市第六届运动会。创作了《太行娘亲》《一代名相陈廷敬》等文艺精品。全力保障迎峰度冬能源供应，既保障了我市群众温暖过冬，又为全省做出了积极贡献。安全生产形势稳定向好，事故起数、死亡人数实现“双下降”。深化平安晋城建设，全年调处各类矛盾纠纷6332件，办理法律援助1129件，人民群众安全感和执法满意度调查实现全省“六连冠”。

　　（五）政府自身建设得到加强。严格落实政府系统全面从严治党主体责任，着力加强“三基建设”，深入推进党风廉政建设和反腐败斗争。坚持依法行政，“七五”普法深入开展，依法完成2274个村（居）委会换届选举工作，积极配合市人大常委会制定了我市首部实体法《晋城市公共交通条例》，严格执行市人大及其常委会的决议决定，主动接受人大法律监督和工作监督、政协民主监督，认真办理市人大代表建议378件、市政协委员提案367件。市县两级行政机构权力清单、责任清单公布运行。“放管服效”改革有力推进，启用首批涵盖17种相关证照的“多证合一”营业执照，在全省率先推行工商注册全程电子化改革，市政务服务中心正式启用，市级“两平台、一张网”建成运行，“13710”信息督办系统高效运转，成为抓落实、促发展的有力抓手。

　　此外，民族宗教、外事侨务、人防、地震、气象、档案、史志、治超、科普、妇女、儿童、老龄、关工委、残疾人、红十字、慈善救助、对口援疆、国防动员和双拥等各项工作都取得了新进展。

　　各位代表，一年来，我们同舟共济，砥砺前行，成绩来之不易。这是习近平新时代中国特色社会主义思想科学指引的结果，是我们认真贯彻执行党中央、国务院和省委、省政府一系列方针政策的结果，是市委统揽全局、正确领导的结果，是市人大、市政协有效监督、全力支持的结果，是全市人民辛勤工作、共同努力的结果。在此，我代表市人民政府，向全市人民，向各民主党派、工商联和无党派人士，向各位人大代表、政协委员，向驻市部队指战员、武警官兵和公安干警，向各位老领导、老同志，向所有关心支持晋城改革发展的各界朋友，表示崇高的敬意和衷心的感谢！

　　在肯定成绩的同时，我们也清醒地认识到，我市发展不平衡不充分问题还比较突出，仍面临诸多困难和挑战。主要表现为：“一煤独大”的产业结构没有根本性改观，经济增速回升主要还是依靠煤炭产业强势拉动，经济总量在中原经济区仍处于第三方阵靠后水平，且呈排位下滑趋势。好项目、大项目少，项目储备不足，非煤产业发展慢、比重小，传统产业优势弱化，新兴产业发展不足。市场化程度较低，体制机制改革滞后，国有企业改革创新能力亟待提高，民营经济活力不足，开放型经济水平不高。经济发展与日趋严格的能源消费管理及资源环境的矛盾更加突出，如何化解环保约束与经济增长的矛盾成为亟待解决的重大课题。基础设施不完善，民生社会事业欠账较多，安全生产基础不牢，社会治理面临一系列新挑战新要求。政府职能转变还不到位，“放管服效”改革亟待深化，有些改革举措和政策落实不力，一些干部工作作风不实、担当精神不够，形式主义、官僚主义不同程度存在。对此，我们一定本着对人民高度负责的态度，采取有力举措，切实加以解决。

二、2018年经济社会发展的主要任务

　　2018年是全面贯彻党的十九大精神的开局之年，是改革开放40周年，是决胜全面建成小康社会、实施“十三五”规划承上启下的关键一年。做好今年政府工作，责任重大，使命光荣。总体要求是，全面贯彻党的十九大及十九届一中、二中、三中全会精神，以习近平新时代中国特色社会主义思想为指导，认真落实全国、全省“两会”精神和市委七届四次全会暨经济工作会议精神，紧扣社会主要矛盾变化，按照高质量发展要求，统筹推进“五位一体”总体布局，协调推进“四个全面”战略布局，坚持稳中求进工作总基调，坚持新发展理念，坚持“供改”与“综改”相结合，围绕打赢防范化解重大风险、精准脱贫、污染防治三大攻坚战，围绕建设全省转型综改示范区的先行区、能源革命排头兵的领跑者、对外开放新高地的桥头堡三大目标，解放思想、凝聚共识、先行先试，奋力开创新时代美丽晋城高质量转型发展新局面。

　　按照总体要求，立足我市全面小康和转型发展阶段性特点，综合考虑速度、效益、结构、动能四个维度的同步协调、相互支撑，确定2018年全市经济社会发展的主要预期目标为：地区生产总值增长7%左右，规模以上工业增加值增长6%，全社会固定资产投资增长10%以上，社会消费品零售总额增长7.5%，一般公共预算收入增长7%以上，城镇居民人均可支配收入增长7%，农村居民人均可支配收入增长7%以上，居民消费价格涨幅控制在3%左右，城镇调查失业率、城镇登记失业率分别控制在6%和4.2%以内。各项约束性指标控制在省下达指标以内。

　　围绕上述目标要求，2018年突出抓好以下十项工作。

　　（一）深入推进产业转型升级

　　晋城有优质的无烟煤和煤层气资源，有悠久的冶铸历史和坚实的制造业基础，有面向中原大市场的独特区位优势，必须立足高质量发展要求，一手抓现有优势放大提升，一手抓新增长点孵化培育，加快新旧动能接续转换，努力构建多元支撑的现代产业体系。

　　深化能源革命。推进煤炭产能置换重组，提高先进产能占比，退出煤炭过剩产能240万吨，重点抓好里必、东大、玉溪等项目。推广“优煤易购”煤炭电子商务销售模式，推进晋城市无烟煤地理标志证明商标办理。巩固燃煤发电机组超低排放改造成果，因地制宜发展风能、太阳能等可再生能源，抓好泽州、陵川、沁水风电项目，进一步优化能源供给结构。加快煤层气开发利用，努力把我市建成抽采先进产能集中区、综合利用引领区、京津冀及中原城市群清洁能源供应区。

　　打造全省先进制造业基地。积极对接“互联网+”和“中国制造2025”，以高端化、智能化、绿色化、服务化为方向，推动制造业优化升级。市财政设立1亿元工业发展专项资金。加快传统产业改造升级。实施现代煤化工升级示范工程，重点抓好晋煤华昱高硫煤洁净利用项目投产运行。推动冶炼铸造产业向园区集聚，重点抓好南村绿色智能铸造创新产业园、晋钢集团智能铸造科技产业园等项目。依托阳城陶瓷工业园区，加快发展中高档建筑陶瓷和日用陶瓷。依托绿洲大麻智能纺织生产线、吉利尔潞绸文化产业园等项目，带动丝麻纺织产业上档升级，让古老的潞绸品牌重放光彩。积极推动军民深度融合创新发展。着力培育壮大新兴产业。光机电行业，紧紧盯住富士康，努力推动其核心技术、核心产业向晋城布局，争取在工业机器人、手机结构件等方面对晋城园区增资扩股10亿元。装备制造行业，依托中船重工、晋煤、兰花等企业的技术和资金优势，着力提升煤机和煤层气装备制造水平，重点抓好中船重工新能源装备制造产业园、清慧轨道交通新型材料结构件等项目。新能源、新材料行业，围绕优势产品，壮大产业规模，着力培育动力电池、太阳能光伏组件、高性能空心玻璃微珠等新产品，重点抓好长征动力三元锂电池、海诺科技二期等项目。生物医药行业，重点抓好海斯制药智能化冻干粉针、兰花中药材深加工等项目。

　　加快发展现代服务业。立足区位优势，主动参与中原城市群物流业分工，统筹规划、科学布局物流园区，加快推进兰花国际物流园区二期、义乌国际商贸城、苗匠电子商务产业园等项目，建设区域性商贸物流中心。吸引各类金融机构来晋城设立分支机构，继续实施企业上市培育工程，用好红土基金、晋城产业转型升级发展基金等各类投资基金，引导金融机构将更多的金融资源配置到实体经济上。与阿里巴巴集团合作，构建全市统一的城市数据中心和政务云平台，加快发展大数据产业，抓好城区中电智云大数据计算中心和泽州华为云计算中心项目。大力发展健康养老、文化体育、居民服务等幸福产业。

　　各位代表，项目是产业转型的基础。今天的项目质量决定了明天的转型质量，今天的项目结构决定了明天的产业结构。我们要按照“转型项目建设年”的要求，以开发区为主战场，以招商引资为重要抓手，建立完善项目推进常态化工作机制，重点抓好总投资1330亿元的68项省市重点工程，带动各县（市、区）、各相关部门全力以赴引进项目、千方百计服务项目，为加快构建现代产业体系夯实基础。

　　（二）奋力打造太行旅游名城

　　晋城气候独特，拥有世界级的自然人文资源，是一块尚未充分开发的旅游宝地。随着交通体系和信息网络的不断完善，旅游成为新的支柱产业，可谓“呼之欲出”、势不可挡，必须积蓄力量、乘势而上，奋力打造太行旅游名城、中国新一线旅游城市。

　　坚持规划引领。聚焦“太行”板块，编制完成《晋城市全域旅游规划》。重点培育以王莽岭为代表的太行风光、以皇城相府为代表的古堡文化、以炎帝陵为代表的农耕文化三大旅游品牌，突出避暑康养特色，全力创建国家全域旅游示范区。积极申报王莽岭世界自然遗产和太行古堡群世界文化遗产。

　　完善基础设施。按照两年建成的目标，加快建设太行一号国家风景道，陵川试验段年内建成，阳城、泽州、沁水、高平都要确定试验路段，全力推进实施。加快建设主城区生态旅游观光绿道，将吴王山森林公园、白马寺山森林公园、司徒小镇、丹河湿地公园四大生态片区及周边分散景区、景点串联起来，形成复合型生态旅游观光带。着力解决主干道至各景区“最后一公里”问题。引进战略投资者，深化旅游体制机制改革，促进重点景区提质升级。注重工业旅游和工业遗产遗址旅游策划，支持引进华谊兄弟星剧场项目，加快建设太行印象文化创意园。鼓励企业和个人挖掘整理、创编研发我市非物质文化遗产产品以及富有地方特色的演艺节目和旅游商品。

　　强化宣传营销。按照国内新一线旅游城市、国际旅游目的地标准，设计城市旅游品牌，策划整体旅游形象。总结推广赴河南开展旅游营销的成功做法，进一步开拓河北、山东、湖北等周边市场。办好第三届海峡两岸神农炎帝文化旅游招商系列活动。举办“夏养晋城旅游养生体验季”等系列活动。创优旅游服务环境，全方位扩大晋城旅游影响力及关注度。

　　（三）扎实推进乡村振兴战略

　　实施乡村振兴战略是新时代“三农”工作的总抓手。晋城的农村小康和新农村建设曾经领先全省、享誉全国。在实施乡村振兴战略中，我们必须再拔头筹，谱写新时代美丽晋城乡村全面振兴新篇章。

　　培育乡村新产业。在保障粮食安全的基础上，着力提升畜牧、蚕桑、设施蔬菜、食用菌、干鲜果、小杂粮、中药材等特色农业发展的质量和水平，重点开发功能食品和药食同源产品，全年新增畜产品1万吨，新增桑园1000亩，改造和新发展设施蔬菜、食用菌设施各1000亩，实施果业提质增效1万亩，杂粮种植面积稳定在70万亩，新种植中药材2万亩。大力发展有机旱作农业，启动“一县两片”省级有机旱作农业示范区建设，创建6个市级有机旱作农业示范片。加大农产品品牌培育力度，推进“三品一标”认证，建设全省重要的有机绿色农产品供给区。支持有条件的乡村探索发展集循环农业、创意农业、农事体验于一体的“田园综合体”。抓好农林文旅康产业融合发展省级试点建设，探索完善功能农业、农旅一体、避暑康养三大模式，2018年重点抓好20个产业融合发展典型。

　　激发乡村新活力。把人力资本开发放在首要位置，加强乡村振兴人才队伍建设。加快培育新型农业经营主体，发展适度规模经营，扶持一批家庭农场、种养大户、农民合作社、社会化服务组织和龙头企业。进一步巩固和完善农村基本经营制度，全面完成农村承包地确权登记颁证工作，为落实第二轮土地承包到期后再延长30年的政策奠定基础。加快推进农村集体产权制度改革，力争年底前80%的村完成清产核资和农村集体经济组织成员身份确认。深化农村宅基地“三权分置”、供销社等改革。

　　塑造乡村新风貌。以农村垃圾、污水治理和村容村貌提升为主攻方向，扎实实施农村人居环境整治三年行动。推进“四好农村路”建设，新改建农村公路757公里。继续开展省市美丽宜居示范村联创活动，创建省级村6个、市级村20个。统筹城乡发展，持续推进巴公、北石店国家级新型城镇化试点及润城、巴公、神农国家级特色小镇试点建设，加强对司徒小镇等非建制镇特色小镇建设的规划和指导。坚持自治、法治、德治相结合，加强农村基层组织建设，推动乡村文化振兴，改善农民精神风貌，确保乡村社会安定有序、充满活力。

　　巩固脱贫攻坚成果。在陵川、沁水两个贫困县脱贫摘帽的基础上，精准施策、坚决完成剩余18个贫困村、近1万贫困人口的脱贫任务。建立健全稳定脱贫长效机制。开展扶贫领域腐败和作风问题专项治理。坚持将扶贫与扶志、扶智结合起来，用心用情用力做好脱贫攻坚各项工作。

　　各位代表，农村情况千差万别。实施乡村振兴战略，必须充分尊重广大农民意愿，科学把握各地差异和特点，精准施策、分类推进，不搞一刀切，不搞统一模式，坚决杜绝形象工程。

　　（四）努力壮大民营经济

　　晋城的发展，民营经济功不可没。民营经济活则晋城活，民营经济转则晋城转，民营经济强则晋城强。我们要像支持国有企业一样理直气壮支持民营企业发展，要像服务招商引资项目一样倾心服务本土企业，再创民营经济新辉煌。

　　营造民营经济发展的良好环境。梳理完善、全面落实我市近年来出台的支持非公有制经济发展的政策举措，坚决破除各种隐性障碍。构建亲清新型政商关系，健全企业家参与涉企政策制定机制。设立中小企业服务中心和民营企业投诉中心，加强对行业协会商会的服务和管理，建立政府与企业家双向沟通机制，及时帮助企业破解各种难题。完善产权保护措施，支持司法机关甄别纠正社会反映强烈的产权纠纷案件。对阻碍民营经济发展的人和事，依法依纪严肃查处。支持各县（市、区）立足优势，突出特色，差异化发展主导产业，切实提高县域经济发展水平。

　　推进“236”行动和“小升规”计划。重点抓好2个旗舰企业、30个行业标杆企业和60个潜力型企业。通过3-5年努力，使旗舰企业销售收入达到100-150亿元，行业标杆企业销售收入达到5-10亿元，潜力型企业销售收入达到2-5亿元，民营经济增加值占GDP比重达到50%以上。今年重点抓好一批民营企业转型实体项目。按照“个转企、小升规、规改股、股上市”路径，选择一批具有发展潜力的小微企业，建立“小升规”企业培育库，对年营业收入1000-2000万元的重点企业开展动态监测、分类指导和精准帮扶。今年重点培育“小升规”企业15户，规模以上工业企业净增10户以上。同时，切实抓好一批服务业“小上限”企业。

　　精心培育优秀民营企业家。激发和保护企业家精神，充分发挥优秀企业家在转型发展中的示范带动作用。建立完善企业家培训体系，支持各县（市、区）与省内外知名高校、培训机构联合开设培训班，对企业家进行针对性、特色化培训。

　　（五）大力实施创新驱动战略

　　晋城作为中部内陆小市，创新资源先天不足，必须用非常之力，下恒久之功，大力实施创新驱动战略，努力营造“鼓励创新、宽容失败”的浓厚氛围，把晋城建成创新之城、活力之城。

　　实施科技创新工程。强化企业技术创新主体地位，引导企业加大研发投入，支持规上工业企业设立研发机构。鼓励企业与省内外高校和科研院所合作，围绕我市支柱产业和新兴产业，着力实施一批重点科技创新项目，促进产学研深度融合。加快市科技研发基地建设，建成富士康纳米光机电研发中心，稳步推进国家级光机电制造业创新中心建设。依托重点企业加快建设一批院士、博士工作站。完善科技成果转化和知识产权交易平台，设立科技成果转化引导基金，让科技成果更多地转化为现实生产力。

　　建设创新创业基地。坚持创新、创业、创投、创客“四创联动”，加强专业化众创空间、创客服务平台和科技企业孵化器建设。对成长型小微企业包容审慎监管。重点支持清控（晋城）打造国家级创新创业基地，抓好高平省级双创示范基地建设，推动开发区申报省级双创示范基地。

　　引进培养各类创新人才。认真落实招商引资、招才引智优惠政策，充分发挥晋城在外商会作用，启动实施“学子归巢”计划，吸引各类人才特别是晋城籍在外成功人士和“双一流”学校学科的毕业生回乡创业。全力支持太原科技大学晋城校区建好优势专业、新增急需专业，健全与我市经济发展密切相关的学科体系。积极推进职业教育资源整合、产教融合，探索建立职教园区。大力弘扬“工匠精神”，充分挖掘本土人才潜力，塑造一批支撑发展的实用型、技能型、创新型人才。加大人才安居保障力度，市政府投资建设44套、近6000平米的高端人才公寓，并通过周转房、公租房、货币补贴等多种形式解决人才住房问题。同时，加大对创新创业、科技研发、成果转化等方面的支持力度，确保各类人才引得进、留得住、用得好。

　　（六）加快建设宜居幸福城市

　　晋城是一座年轻的城市，也是一座具有深厚文化底蕴的千年古城。提升城市品质既是老百姓的殷切期盼，更是政府义不容辞的责任。我们要坚持以人为核心，不断营造宜居环境，提升幸福指数，进一步增强晋城在山西和中原城市群的影响力。

　　抓好顶层设计。按照“拓展、改造、提质、传承”的要求，高起点修编城市总体规划，扎实开展全国城市设计试点工作，加快推进“多规合一”，切实发挥规划的引领和管控作用。启动行政区划调整工作，着力解决制约我市长远发展的“一市一区”问题。

　　启动老城更新与保护。坚持“延续文脉不乱拆滥建，尊重规律不复古重建，严格管控不放任自流，稳中求进不急于求成”的原则，在保护城市肌理、传承历史文脉的基础上，对两平方公里老城内的基础设施进行更新改造、居住环境实施综合整治，到2020年，基本解决老城交通拥堵、河道阻塞、设施老化等突出问题，全面改善老城环境，让老城居民生活得更加方便、舒心，更好地分享改革发展成果。今年，在完成规划编制的基础上，重点实施南大街立面改造工程，恢复历史建筑风貌；完成新市西街、南大街地下管网及路面改造；启动景德桥、景忠桥两大片区棚户区改造及东河、西河沿线治理工程；加快推进东西大街改造前期工作；全力做好改造范围内的房屋征收补偿工作。

　　加快金村新区建设。坚持高起点规划、高标准建设、高水平推进，结合高铁、机场及文教园区建设，拓展城市发展空间，着力打造全面体现新发展理念，集教育科研、生态绿屏、交通枢纽、旅游集散、区级行政中心等功能为一体的低碳智慧城、生态宜居城、科技创新城。今年，在完善规划修编及城市设计的基础上，加快金村大道南延、北延等骨干路网建设，推进丹河龙门湿地公园二期、三期和高铁站前可视山体绿化工程，做好晋城东站、站前广场、站前公园以及高铁站与市区快速连接通道的设计、征地等前期工作。

　　实施“三通”工程。重点抓好总投资72.16亿元的25项工程。通道工程，主要解决中心城市区域交通瓶颈问题，重点建设陵沁路西北环改线、中原街改造及综合管廊建设、畅安路北延、白水街西延等项目。通畅工程，主要解决城市断头路和街路互通问题，重点建设景西南路南延、黄华街南延、207国道改线互通工程、高速东口改造等项目。通顺工程，主要通过对既有城市道路修补更新，方便群众出行。

　　提升城市管理服务水平。持之以恒推进全国文明城市创建。做好国家卫生城市复审迎检工作，力争两年内实现国家卫生县城全覆盖。有计划、有步骤地推进“城市双修”工作。加快建设智慧城市，实现城市管理的信息化和智能化。深入开展市区“双违”整治和“三洗”“六见”专项行动。“双违”整治，保证市区违法占地、违法建设“零增长”，新建道路两侧全面“清零”。“三洗”“六见”，即通过开展洗地、洗树、洗墙三项行动，实施开门见绿、游园见景、空地见翠、道路见树、逢山见林、河清见底六大工程，进一步净化、绿化、美化市区环境。设立流动红旗，在市区各机关、企事业单位、镇（街道）、村（社区）开展竞赛评比，推动“双违”整治和“三洗”“六见”专项行动取得实实在在的效果。同时，着力解决好市区“停车难”“买菜难”等突出问题。

　　（七）持续深化重点领域改革

　　我市重点领域新一轮改革刚刚起步、尚未破题，迫切需要我们既敢于担当，又本领高强。“允许改革有失误，但不允许不改革”。要坚持问题导向，聚焦关键环节，既敢于出招又善于接招，努力跻身全省改革第一方阵。

　　深化开发区改革创新。对标转型综改示范区，加快晋城经济技术开发区“一区三园”整合扩区进度，深化“三化三制”改革，加大基础设施、标准厂房建设和招商引资力度，加快建设台商投资产业园，积极谋划中外合作（国际）产业园。推动高平开发区全面落实改革方案，推动阳城、沁水省级开发区和陵川省级生态文化旅游开发区尽快获批。坚持“一次规划、滚动开发”，有序推进各开发区土地转用和征收审批，做好“九通一平”，确保入区项目“熟地”供应。2018年，晋城经济技术开发区完成土地收储3000亩。优先在开发区布局产业转型项目，工业项目一律进园入区。

　　深化国企国资改革。推动晋城市国有资本投资运营公司规范运作，重点抓好优化资本布局、分类改革、专业化整合重组等工作，出资企业由42户减少到30户以下。积极开展混合所有制改革试点。推动兰花集团聚焦主业、瘦身健体，提升融资能力，加快产业转型。推动天泽集团优化股权结构，全面启动上市工作。积极稳妥地推动“僵尸”企业市场出清。加快解决国有企业办社会等历史遗留问题，全面完成国企职工家属区“三供一业”分离移交。支持省、市、县三级国有企业联动发展，鼓励同行业优化重组和上下游联营。

　　深化“放管服效”改革。深入开展“六最”营商环境建设年活动。全市域推开企业投资项目承诺制改革试点。在开发区推行“证照分离”改革试点。大力推进综合执法机构机制改革，着力解决执法不规范、不到位等问题。清理群众和企业办事的各类证明，没有法律法规依据的一律取消。抓好高平市相对集中行政许可改革省级试点，及时总结经验，借鉴外地做法，在市县两级全面推开。

　　积极争取并全力抓好改革试点试验。深入贯彻国务院42号文件精神，认真落实市委确定的重点改革任务，先行先试、以改促转。积极争创国家级资源型经济转型发展金融改革创新试验区。全力争取国家和省里对煤层气资源税改革的支持。总结泽州县国家级农村集体经营性建设用地入市试点经验，争取在各县（市、区）尽快推开。

　　（八）着力扩大对外开放

　　晋城地处山西一隅，最大的不足是开放的不足。但站在中原经济区及更大的范围来看，却具有对外开放的独特优势和巨大潜力。我们必须以全新的视野拓展开放空间，加强区域合作，全力建设我省内陆地区对外开放新高地的桥头堡。

　　积极融入国家战略。发挥中原城市群核心发展区城市的比较优势，以晋郑、晋焦城际公交开通为起点，切实加强与中原城市群其它城市间在交通、产业、人才交流、科教文化等方面的深度合作。积极对接京津冀、长三角、珠三角、港澳台和“一带一路”国家重点战略区域，找准结合点，有针对性地开展交流合作，为转型发展开拓更加广阔的空间。

　　全力构建开放通道。加强晋城中长期综合交通体系前期研究谋划。加快太焦高铁晋城段建设，做好呼（和浩特）南（宁）高铁豫西通道对接工作。有序推进晋城机场前期工作，开工建设阳城通用机场。加快阳蟒高速建设。启动晋城-阳城高速公路八车道拓宽改造工程。积极推动跨市的陵川-长治一级公路，阳城-运城、沁水-安泽高速公路前期工作。加快推进陵川王莽岭-新乡、阳城蟒河-济源出省口高速公路建设。抓好阳济线交通部重载交通示范项目。

　　着力打造开放平台。继续做好中国（山西）自贸试验区晋城片区的申报设立工作。促进兰花保税物流中心与郑州铁路局、晋城北站深度合作，加快晋城铁路口岸建设。谋划晋城航空口岸建设。推动建立晋城至天津港物流专线。

　　加大招商引资力度。坚持引资与引智引技并重，研究完善我市招商引资引智优惠政策。按照“系统定向精准”的要求，制作“招商地图”，依托富士康、中船重工、晋煤等重点企业，以企招商、以商招商、以产业链招商，千方百计引进一批大项目、好项目。

　　（九）精心擦亮晋城生态品牌

　　生态是晋城的优势、晋城的品牌，也是晋城永续发展的巨大财富。我们要牢固树立“绿水青山就是金山银山”的理念，坚持保护与利用并重，切实把环境污染治理好，把绿水青山呵护好，把能源资源利用好，为人民群众创造良好的生产生活环境。

　　从严治理环境污染。巩固扩大中央、省环保督察反馈问题整改成效，坚决打好大气、水、土壤污染防治攻坚战。突出抓好大气污染防治。按照环保部对京津冀大气污染传输通道“2+26”城市要求，充分利用高科技手段对污染排放精准监测、溯源，对雾霾成因深度解析研究，科学制定雾霾治理方案。狠抓“控煤”“治污”“管车”“降尘”4个关键环节，深入开展大气污染综合治理，精准实施重点行业错峰生产、错峰运输和重污染天气应急减排措施，推动环境空气质量持续改善。深化水污染防治，坚持“五水同治”，全面落实“河长制”，探索实施“河警长制”，加快污水处理和人工湿地重点项目建设，严格落实畜禽养殖禁养区管理要求，强化沁（丹）河流域生态修复与保护，加大张峰水库饮用水源保护力度。全面消除市区黑臭水体。加强土壤污染防治，开展土壤污染状况详查，实施农用地土壤环境分类管理，建立污染地块动态清单和联动监管机制。

　　持续改善生态环境。大力推进国土绿化，全市完成营造林工程2.32万亩，中幼林抚育2.8万亩。加快环城生态圈建设和环城破损山体修复，重点抓好环市区8个森林公园（湿地公园）以及重点道路、河流沿线的绿化工程。以建设“家门口的公园”为重点，新建文昌游园、春晓游园、文博路游园、太岳街游园4个城市小游园，切实抓好新建城市道路绿化美化，持续开展“微绿化”升级行动。做好国家森林城市复审迎检工作。

　　推动绿色低碳发展。持续推进国家级低碳试点市和循环经济示范市建设，引深“六个一”低碳示范工程。实施能源消费总量和强度双控行动，加强节能重点工程建设，推进工业固废减量化、资源化、无害化。建立完善生态文明体制机制。

　　（十）倾力抓好民生社会事业

　　人民对美好生活的向往是我们始终不渝的奋斗目标。我们要牢固树立以人民为中心的发展思想，抓住人民群众最关心最直接最现实的利益问题，既尽力而为，又量力而行，不因事难而推诿，不因善小而不为，一件接着一件办，一年接着一年干，切实增强全市人民的获得感、幸福感、安全感！

　　大力推动教育提质。编制《晋城教育现代化2035》。推进县域内城乡义务教育一体化改革发展，实施学前教育、特殊教育、高中教育优质提升计划，努力实现教育资源均衡配置。推进职业教育产教融合和学徒制试点。试行民办学校规范分类管理。着力解决“入园贵”、“择校热”、中小学生课外负担重等问题。加强师资队伍和师德师风建设。加快推进东南新区学校建设，确保凤城中学高中部、市中小学综合实践基地、晋城中专装备制造实训楼按时投入使用。

　　加快建设健康晋城。深化县乡医疗卫生机构一体化改革，推动县级医疗集团规范运行，深入推进分级诊疗、家庭医生签约服务和医联体建设，推动优质医疗资源下沉。健全完善公立医院现代管理运行新机制。提高基本医保和大病保险保障水平。加强疾病预防控制和食品药品监管工作。广泛开展全民健身运动。加快推进市人民医院易址新建。

　　积极促进就业创业。全面提高国家级创业型城市建设水平。鼓励职业院校和企业深化校企合作，充分实现毕业入企和进校提升的双向融合。统筹做好高校毕业生、农村转移劳动力、城镇失业人员、4050人员、退役军人等重点群体的就业工作。努力提高城乡居民收入水平。

　　强化民生兜底保障。实施全民参保计划，继续提高退休人员基本养老金和城乡居民基础养老金，健全被征地农民基本养老保险制度，推进失业、工伤、生育保险覆盖规定职业人群。合理调整社会最低工资标准。逐步提高城乡低保、社会救助、抚恤优待等标准。积极应对人口老龄化，大力提高养老服务水平。做好军烈属优抚、防灾减灾救灾工作。加强残疾人康复服务。支持公益慈善事业发展。

　　提升住房保障水平。认真落实新三年棚改攻坚计划，新开工建设各类棚户区住房6764套，其中城区4673套。加大房地产用地供应管控，建立健全房地产市场平稳健康发展的长效机制。推进住房制度改革，加快发展住房租赁市场。扩大住房公积金制度覆盖面，更好满足群众购房需求。抓好房地产市场监测，大力整顿规范房地产市场秩序。

　　完善公共文化服务体系。培育和践行社会主义核心价值观，深入实施公民道德建设工程。大力推进文化惠民，继续开展文化科技卫生“三下乡”、农村公益电影放映等活动。完善公共文化基础设施，加大政府购买公共文化服务力度，丰富群众文化生活。加强文艺创作，推出一批精品佳作。加强红色遗迹、战斗遗址的保护，加强非物质文化遗产保护传承。大力实施文明守望工程和乡村文化记忆工程。积极创建国家公共文化服务体系示范区。

　　深入推进“厕所革命”。制定“厕所革命”行动计划。主城区要在国家卫生城市复审之前全部取缔或改造公共旱厕，各县城公共旱厕两年内“清零”，旅游景区年底消灭旱厕，因地制宜推进农村无害化卫生厕所建设和改造。

　　防范化解重大风险。以“四铁”要求狠抓安全生产，坚决遏制重特大事故。优化政府债务管理，积极防范和化解企业债务违约风险，依法严厉打击各类金融犯罪和非法集资活动。加强和创新社会治理，扎实推进扫黑除恶专项斗争，健全社会治安防控体系，完善多元化纠纷解决机制，提升平安晋城建设水平。

　　此外，还要切实做好民族宗教、外事侨务、人防、地震、气象、档案、史志、治超、科普、妇女、儿童、老龄、关工委、残疾人、红十字、慈善救助、对口援疆、国防动员和双拥等工作，为我市改革发展凝聚强大合力。

　　在做好以上工作的同时，今年市政府还要为民办好10件实事：一是实施2400余名残疾预防重点干预和残疾儿童抢救性康复项目；二是为全市城乡怀孕妇女提供免费产前筛查和诊断服务；三是新建60个农村老年人日间照料中心；四是免费送戏下乡700场；五是实施全民技能提升工程，培训农村进城务工人员32000余人、城镇失业人员7200余人；六是为1000例贫困白内障患者免费进行复明手术；七是实现农村寄宿制学校后勤保障全覆盖；八是在大型农产品批发市场、农贸市场和超市建立10个“食品安全快速检测室”；九是解决全市具备通车条件但尚未通车的579个建制村老百姓的出行问题；十是培育新型职业农民1000人。

　　各位代表，我们将信守承诺，坚决把好事办好、实事办实，向全市人民交上一份满意的答卷。

三、全面加强政府自身建设

　　当今时代，地区之间的竞争不仅是基础设施等硬实力的竞争，更是营商环境等软实力的竞争，核心是政府服务能力的竞争。我们要转变思想观念，全力建设人民满意的服务型政府，以软实力的优势带动硬实力的提升。

　　加强政治建设。树牢“四个意识”，增强“四个自信”，自觉用习近平新时代中国特色社会主义思想武装头脑、指导实践、推动工作，始终在思想上政治上行动上同以习近平同志为核心的党中央保持高度一致。严格落实全面从严治党主体责任，切实加强政府系统党的建设，深入推进“两学一做”学习教育常态化制度化，扎实开展“不忘初心、牢记使命”主题教育，持续加强“三基”建设，以党建引领改革发展各项工作。

　　严格依法行政。严格遵守宪法法律，严格执行人大及其常委会的决议决定，认真办理人大代表建议、政协提案，主动接受人大和政协的监督。积极开展政府立法工作，并适时向市人大及其常委会提出地方立法建议。做好规范性文件备案审查工作。坚持依法决策，完善重大行政决策公众参与、专家论证、合法性审查及实施后评估等制度。强化行政执法监督，做好行政复议和行政应诉工作。加强审计监督，在各级政府及其工作部门全面推行内审制度。加强智库建设。发挥好政府法律顾问作用。在政府部门推行“两公开一评议”工作，即政务公开、财务公开，接受人大和社会评议，干得好不好要看实际效果、要让群众来评判。

　　加快转变职能。按照国家和省统一部署，有序推进政府机构和事业单位改革，确保改革过程中思想不乱、队伍不散、工作不断、干劲不减。全面推行“双随机、一公开”监管。完善市县两级“两平台、一张网”，全面推行“两集中两到位”“一颗印章管审批”，加快推进“最多跑一次”改革，让数据“多跑路”，群众和企业少跑腿，实现“在晋城办事不找关系”。把为项目建设营造良好环境作为转变政府职能的重要抓手，严厉整治干扰阻挠重点工程建设的行为，对普通群众耐心教育开导，对党员干部坚持挺纪在前，对带有黑社会性质的组织依法严厉打击。

　　勇于担当任事。牢固树立“为人民干事是天职、不干是失职”的理念，大干快干、苦干实干，在推动转型发展中干出实打实的新业绩，干出群众的好口碑。加强对政府工作人员的教育培训，增强“八项本领”，做到“五个过硬”。全面深化、拓展、延伸“13710”信息化督办制度，对懒政怠政者严肃问责，重点整治表态多调门高、行动少落实差等形式主义、官僚主义新表现，破除“中梗阻”，增强执行力。认真落实省委、市委激励干部担当作为干事创业办法、支持干部改革创新容错办法，旗帜鲜明给积极干事者撑腰鼓劲，努力营造“晋城的事大家想、大家说、大家干”的浓厚氛围。

　　恪守廉政底线。市政府一班人要发挥“头雁效应”，以身作则、以上率下，坚决纠正“四风”突出问题，严格执行中央八项规定实施细则及省、市实施办法。坚持用制度管权管事管人，加强对重点领域、关键环节、重要岗位权力运行的监督制约，着力构建廉政风险防控长效机制。规范财政收支管理，严控“三公”经费，真正把有限的财政资金花在群众的心坎上，花在为晋城打基础、利长远上。

　　各位代表，新时代要有新气象，更要有新作为。让我们更加紧密地团结在以习近平同志为核心的党中央周围，在市委的坚强领导下，以永不懈怠的精神状态和一往无前的奋斗姿态扎实做好各项工作，为开创新时代美丽晋城高质量转型发展新局面而努力奋斗!
